# Validation and Cultural Adaptation of Persian Version of Multidimensional Health Assessment Questionnaire in Rheumatoid Arthritis Patients

**DOI:** 10.31138/mjr.130723.pvm

**Published:** 2024-01-08

**Authors:** Elham Aflaki, Faezeh Sehatpour, Sheida Banihashemi

**Affiliations:** 1 Autoimmune Disease Research Centre, Shiraz University of Medical Sciences, Shiraz, Iran,; 2 Department of Internal Medicine, Shiraz University of Medical Sciences, Shiraz, Iran,; 3 Department of Community Medicine, Shiraz University of Medical Sciences, Shiraz, Iran

**Keywords:** rheumatoid arthritis, multidimensional health assessment questionnaire, validity, reliability

## Abstract

**Background:**

Rheumatoid arthritis (RA) is a multidimensional disease. In addition to quantitative factors, qualitative factors play an important role in the progress and outcome of the diseases. One of the most effective methods of collecting qualitative information is questionnaires reported by patients. The data obtained from the questionnaires are as important as the clinical criteria. Multidimensional health assessment questionnaire (MDHAQ) is one of the latest questionnaires that provide useful information in a short time.

**Objectives:**

To investigate the reliability and validity of the Persian form of MDAHAQ for the use of Iranian patients.

**Method:**

Two groups of participants were selected for this study. The validity test group included 110 patients, and the reliability test group included 140 patients. Translation and adaption of MDHAQ were performed by using Guillemin guidelines. The reliability was tested by using test-retest and Cronbach’s alpha for internal consistency. Persian version of the health assessment questionnaire (HAQ) was used for assessing the criterion validity. The correlation between the MDHAQ score and Disease Activity Score-28 (DAS28), Clinical Disease Activity Index (CDAI), and the Persian version of the health assessment questionnaire (HAQ) was evaluated using the Spearman coefficient. Discriminant validity was tested in groups of patients based on two varied disease activities based on CDAI and DAS28.

**Results:**

Test-retest with intra-class correlation coefficient (ICC) gave a coefficient of 0.865(95% CI: 0.809, 0.904) for physical function and 0.786(95% CI: 0.698, 0.848) for psychological items. Cronbach’s alpha was 0.885 and 0.705 for physical function and psychological dimensions respectively. The Persian version of the MDHAQ had a good to strong correlation with the Persian version of the HAQ (ranging from 0.604 to 0.962) and also with CDAI (ranging from 0.616 to 0.838) and a moderate correlation with DAS28 (ranging from 0.415 to 0.439).

**Conclusion:**

The Persian form of MDHAQ is a reliable and valid instrument for the routine evaluation of RA patients in rheumatology clinics in Iranian RA patients.

## INTRODUCTION

Rheumatoid diseases, such as rheumatoid arthritis (RA), are multifactorial diseases. RA is one of the most common inflammatory and destructive arthropathies in the world.^1^ Unlike other chronic diseases, quantitative measurements alone cannot be considered the golden standard for following and determining the prognosis of patients with rheumatoid diseases over time.^2^ Qualitative factors such as pain, fatigue, functional disability, and psychological problems play an important role in the prognosis of rheumatoid diseases and should be considered as an essential factor in the treatment of patients with rheumatoid arthritis.^3,4^

Qualitative assessment of patients with RA has developed extensively over the past three decades. One of the most effective methods for collecting qualitative information is self-reporting patient questionnaires. Information obtained from the questionnaires is just as useful as clinical evaluations, laboratory tests, and radiographic findings when predicting functional disability, occupational constraints, illness costs, and early mortality in patients with RA. Patient questionnaires should be included along with vital signs at each clinic visit.^5,6^

Currently, several questionnaires have been designed to measure the outcome of rheumatologic diseases. The most commonly used questionnaire for patients with RA is the Health Assessment Questionnaire (HAQ), which is available in Persian, and the Multidimensional Health Assessment Questionnaire (MDHAQ), which is not yet available in Persian.^2^ Although the HAQ and MHAQ questionnaires were useful in rheumatic diseases, especially rheumatoid arthritis, there were two major problems associated with these questionnaires. HAQ questionnaire did not directly consider the mental condition of patients, which is one of the most important factors when it comes to a patient’s recovery. The scoring system was also very simple; many patients who had scores within the normal range also experienced significant functional limitations.^2,7^

MDHAQ is one of the latest questionnaires designed for use in Rheumatology clinics. It has been translated and validated in many different countries and can be used for patients of different cultures and languages.^8^ This questionnaire provides crucial information about important dimensions of rheumatologic diseases while being filled out in a short amount of time. The psychological status of patients is also evaluated in the MDHAQ, which is not reviewed in other questionnaires.^9–11^

The MDHAQ scoring system is also more accurate compared to previous questionnaires and is more practical for use in busy rheumatology clinics.^12, 13^

According to previous studies, the original version of MDHAQ has acceptable validity and reliability.^2^ Unfortunately, we do not have useful questionnaires for routine evaluation of qualitative factors of RA in Iranian patients. Therefore, due to the importance of this issue, we decided to translate this questionnaire and evaluate its validity and reliability for assessing the severity of disease and quality of life of patients with RA.

## METHOD

### Study subjects and setting

This study included Persian-speaking patients with RA who were referred to the Motahari Clinic of Shiraz University of Medical Sciences from April to June 2022. Each participant who was at least 18 years old and fulfilled the criteria of the American College of Rheumatology for RA patients^14^ was recruited in this survey. To evaluate the validity and reliability of the Persian version of the MDHAQ (MDHAQ-P) questionnaire, two groups of participants were selected for this study. The validity test group included 110 patients, and the reliability test group included 140 patients. Before beginning the questionnaire, a clinician explained the goals of the study and how to complete the questionnaire. Patients willingly participated in this survey, and verbal consent was obtained. This study was approved by the ethics committee of Shiraz University of Medical Sciences (SUMS) contract number of IR.SUMS.MED.REC.1398.248.

### Exclusion Criteria

Participants who had a history of cognitive impairment, any type of neuromuscular diseases, drug addiction, patients with morbid obesity, and those who declined participation were excluded from this study.

### Tools used

We used both MDHAQ-P and the Persian version of the Health Assessment Questionnaire (HAQ), which was previously validated by Rastmanesh et al.^15^ In this study, we also utilised erythrocyte sedimentation rate (ESR), Disease Activity Score-28 (DAS28) and Clinical Disease Activity Index (CDAI) to assess the clinical value of MDHAQ-P.^16,17^

### MDHAQ

The MDHAQ is a 2-page questionnaire that consists of 10 parts: physical function (FN), psychological status (PS), pain, Rheumatoid Arthritis Disease Activity Index (RADAI), patient’s global health status estimate (PTGL), symptom checklist review of system (ROS), morning stiffness (AM), change in status over the last week (CHG), exercise habits (EX), fatigue (FT) and recent medical history.

The first part of MDHAQ includes two sections: FN and PS. FN includes 10 items (question (Q) 1. a–j) about activities of daily living and is scored between 0–3 (0 = without any difficulty, 1 = with some difficulty, 2 = with much difficulty and 3 = unable to do). Finally, the sum of the raw score is divided by three, giving the score between 0–10. Three items (Q 1. k–m) about the quality of sleep, depression, anxiety, and stress constitute the PS part. Each part of the PS section is scored 0–3.3(0 = without any difficulty, 1.1 = with some difficulty, 2.2= with much difficulty, and 3.3 is unable to do) for a total of 0–9.9. PS dimension is not calculated in the overall score.

The second part is about the patients’ pain due to the underlying disease (RA). The patient is asked to rate his pain from 0–10 by using a visual analog scale (VAS) including 21 circles which are separated in 0.5 units and a total score of 0–10.

RADAI (Q 3) includes a group of joints in which the patient scores the associated joint pain on a scale of 0–3. Neck pain and lower back pain are also recorded in this section but are not included in the overall scoring.

PTGL (Q 4) is about the patient’s attitude toward the disease and how the disease affects their health. The patient scores their health status on a scale of 0–10 with a score of zero indicating complete satisfaction with their health status. Unusual fatigue is also scored between 0–10 in the FT dimension.

ROS (Q 7) is a quantitative checklist of the patient’s systems over the last month and scoring includes the number of symptoms on the checklist. AM (Q 6) is rated by yes or no and scored in the number of minutes, with a maximum of 300 minutes. CHG (Q 7) is scored from 1–5 (1 =much better, 2 =better, 3 = same, 4 =worse, 5 = much worse). In Q 8, EX, the frequency of aerobic exercises for at least 30 minutes is scored from 0–3 (3 = 3 or more times a week, 2 = 1–2 times per week, 1 =1–2 times per month and 0 = don’t exercise regularly). A score of 9 is assigned to the patients who can’t exercise due to disability/handicap users.

Recent medical history, which is the last section of the questionnaire, is not scored quantitatively.

The Routine Assessment of the Patient Index Data 3 (RAPID3) is used for scoring. Scores range from 0–30 and are then classified into four categories (very severe < 12, moderate- intensity 12–16, low- intensity 3.6 and recovery 3 ≤). This score is obtained from the sum of the scores from the first part, which includes FN and PS, pain, and PTGL.^18^

#### DAS28

DAS28 measures disease activity and describes the severity of the disease by using clinical and laboratory data. It consists of four components: number of swollen joints (out of the 28 joints), number of tender joints (out of the 28 joints), ESR, global patient pain, and health status estimation. This is then classified into four categories: high disease activity (DAS28>5.1), moderate activity (DAS28= 3.21, 5.1), low disease activity (DAS28= 2.6, 3.2), and in remission (DAS28 < 2.6).^19^

#### CDAI

CDAI is another quantitative index for evaluating disease activity. It consists of four components: number of the swollen joints (out of the 28 joints), number of the tender joints (out of the 28 joints), the patient’s general estimation of his pain and health status, and the physician’s general estimation of the patient’s health status. This index is also classified into four categories: high disease activity (CDAI= 22.1–76), moderate activity (CDAI=10.1–22), low disease activity (CDAI= 2.9–10), and in remission (CDAI<2.9).^20^

### Translation and modification of Persian version of MDHAQ

The translation and modification of the Persian version of MDHAQ (MDHAQ-P) was done according to Guillemin and his colleagues’ guidelines.^21^ After obtaining permission from the original writer, the original version of MDHAQ was independently translated into Persian by two translators who were fluent in English and native Persian. Both translators reached an agreement in one session. Back translation of the Persian version of the questionnaire into English was done blindly by two different independent translators. Items that were not widely used in Iranian culture were modified by Iranian lifestyle and culture during cross-cultural analysis. The final Persian version of MDHAQ was reviewed by a committee of four expert rheumatologists. They commented on scoring, grammar, and the necessity of certain parts of the questionnaire; modifications were made based on their comments.

The Persian version of the questionnaire was given to 10 patients during a pilot study to check for face validity. They were asked about how much of the questionnaire they comprehend, and whether or not they understand the relevancy between each question and their corresponding items. Modifications were then made to parts of the questionnaire that patients said sounded ambiguous. In the translation and adaptation process, the ROS section moved from part 5 to part 9 of the MDHAQ-P. According to recommendations from rheumatologists in the review committee and feedback from participants in the pilot study, in the section regarding the recent medical history (Q10), items on ethnicity, medical insurance, date of birth, weight, and height were omitted.

### Reliability of the MDHAQ-P

The internal consistency of MDHAQ-P items was evaluated using Cronbach’s alpha. Reliability was also tested with the test–retest, and the coefficient of repeatability was calculated within a 2–3week interval. Due to the multiple dimensions of MDHAQ, only the reliability of the two first dimensions (FN and PS) was evaluated in current and similar studies.

### Validity of the MDHAQ-P

The validity of the MDHAQ-P was estimated by comparing its scale to the HAQ disability index (HAQ-DI) which is derived from the Persian version of HAQ. For assessing the discriminate validity, the patients were categorised into two groups based on RA activity: patients whose RA was inactive (whose CDAI score was ≥10 and DAS28 ≥3.2) versus patients whose RA was active (CDAI> 10 and DAS28>3.2).

### Statistical Analysis

We estimated the proportions and means ± standard deviations (SD) to describe the data. Internal consistency of the MDHAQ-P was assessed using Cronbach’s alpha. Interclass correlation coefficient (ICC) was considered for the estimation of reproducibility of the MDHAQ-P. Cronbach’s alpha and ICC>0.7 indicate strong reliability, ICC 0.4–0.7 indicates good reliability, and ICC < 0.4 indicates moderate to poor reliability for MDHAQ-P. Spearman rank order coefficient was used to evaluate the association among MDHAQ-P, HAQ, and HAQ DI. The correlation of MDHAQ-P and clinical values was also measured using the Spearman coefficient. Spearman coefficient (rho) > 0.7 indicates a very strong correlation, rho 0.5–0.7 indicates a strong correlation, rho 0.3–0.5 indicates a moderate correlation, and rho <0.3 indicates a weak correlation. A Non-parametric test was used for comparison of the average scores of MDAHQ-P items in patients whose RA was active and patients who were in remission. The significance level was set at 0.05. SPSS software version 21 was used for this data analysis.

## RESULTS

### Validity

Out of the 110 RA patients asked to participate in the study, 100 patients agreed to participate and were placed in the validity group of the study (90.91%). A total of 100 patients, 84 women (84%) and 16 men (16%), filled out the questionnaires. Detailed clinical characteristics of the participants and mean scores (SD) on every MDHAQ-P item are shown (**Table 1**).

**Table 1.** Clinical characteristics of the participants and characteristics of scores of MDHAQ-P items.

**Variables**	**Number**	**Mean±SD**	**Median**	**Mode**	**MIN, MAX**
HAQ-DI^a^	100	0.41± 0.46	0.25	0	**0,1.75**
CDAI^a^	99	6.58± 5.63	5.5	2	**0, 23**
DAS28^a^	89	2.41± 0.96	2.49	1.75	**0, 4.65**
ESR^a^ mm/h	89	19.06± 14.67	15	9	**0, 101**
	**MDHAQ-P components:**				
FN^a^	100	1.75± 1.93		0	**0,7**
PS^a^	100	2.28± 2.25	1.65	0	**0,8.8**
PN^a^	100	3.26± 2.63	3	0	**0,10**
JCTC^a^	99	1.34± 1.65	0.8	0	**0,7.7**
PTGL^a^	100	3.22± 2.38	3	0,3	**0,10**
FT^a^	100	4.24±2.64	5	6	**0,10**
RAPID3	100	8.09± 6.06	8.15	0	**0,25.7**

RAPID3: Routine Assessment of Patient Index Data3; HAQ-DI: Health Assessment Questionnaire Disability Index; CDAI: Clinical Disease Activity Index; DAS28: Disease Activity Score-28; ESR: Erythrocyte Sedimentation Rate; FN: Functional status; PS: Psychiatry status; PN: pain; JCTC: self-report joint count; PTGL: patient global health status; FT: fatigue; ROS: review of system.

The mean age of the patients was 55 ± 7.09 years. The prevalence of morning stiffness was 41% among the participants. The average duration of morning stiffness was 9.05 ± 58.73 minutes. 36% of the participants reported better conditions in comparison to the previous week. 35% reported no change in their condition, 19% reported worsened condition, and 10% reported improvement in their condition, respectively. In section EX, the most selected item on the questionnaire was related to not exercising regularly (74%), and the item that received the fewest marks belonged to exercising three or more times a week (4%). Based on the RAPID3 category, 25% of the participants were in remission, 14% were in the low severity category, 33% were in the moderate severity category and 25% were in the high severity category. RAPID3 and its components had a good to strong correlation with the Persian version of the HAQ (ranging from 0.604 to 0.962) and also with CDAI (ranging from 0.616 to 0.838) and a moderate correlation with DAS28 (ranging from 0.415 to 0.439). EX had no significant correlation with any of the disease activity parameters (**Table 2**).

**Table 2.** Correlation between the MDHAQ-P and Persian versions of the HAQ, CDAI, and DAS28.

	**FN** ^**a**^	**PS** ^**a**^	**PN** ^**a**^	**JCTC** ^**a**^	**PTGL** ^**a**^	**MS** ^**a**^	**CHG** ^**a**^	**FT** ^**a**^	**ROS** ^**a**^	**RAPID3** ^**a**^
**HAQ-DI** ^**a**^	0.962^*^	0.331^*^	0.527^*^	0.402^*^	0.604^*^	0.475^*^	0.393^*^	0.314^*^	0.325^*^	0.733^*^
**CDAI** ^**a**^	0.616^*^	0.447^*^	0.799^*^	0.639^*^	0.809^*^	0.402^*^	0.435^*^	0.533^*^	0.334^*^	0.838^*^
**DAS28** ^**a**^	0.414^*^	0.263^**^	0.424^*^	0.401^*^	0.415^*^	-	-	-	-	0.439^*^

HAQ-DI: Health Assessment Questionnaire Disability Index; CDAI: Clinical Disease Activity Index; DAS28: Disease Activity Score-28; FN: Functional status; PS: Psychiatry status; PN: pain; JCTC: self-report joint count; PTGL: patient global health status; MS: morning stiffness; CHG: change in health status last week; FT: fatigue; ROS: review of system; RAPID3: Routine Assessment of Patient Index Data3.

* p<0.01,

** P<0.05.

Mean scores of different parts of MDHAQ-P, except for EX, were significantly different between the participants who were active with RA and those who were in remission based on CDAI. Mean scores were significantly different in RAPID3 components, JCTC, and CHG in the two groups categorized based on DAS28 (**Table 3**).

**Table 3.** Discriminate validity of MDHAQ-P with RA activity indexes.

	***CDAI*** ^**a**^			***DAS28*** ^**a**^						

**Items**	**≥10**	**>10**	**p-value**	**≥3.2**	**>3.2**	**p-value**
**FN** ^**a**^	1.15± 1.48	4.11± 1.77	<0.001	1.30± 1.72	3.07± 2.24	<0.001
**PS** ^**a**^	1.86± 2.15	3.85± 1.97	<0.001	1.90± 2.09	3.42± 2.50	0.010
**PN** ^**a**^	2.63±2.38	5.96±1. 80	<0.001	2.46± 2.28	5.89± 1.91	<0.001
**JCTC** ^**a**^	1.04± 1.46	2.62± 1.84	<0.001	1.09±1.56	2.29± 1.81	0.006
**PTGL** ^**a**^	2.38± 1.72	5.75± 2.13	<0.001	2.5± 2.07	4.67± 2.03	<0.001
**MS** ^**a**^	1.18± 0.69	1.70± 0.47	0.002	1.17± 0.70	1.50± 0.62	0.058
**CHG** ^**a**^	2.49± 0.86	3.20± 0.89	0.004	2.52± 0.84	3.06± 1.00	0.048
**EX** ^**a**^	1.42± 3.00	1.60± 3.28	0.823	1.41±3.01	1.67± 3.41	0.772
**FT** ^**a**^	4.14 ± 2.97	6.07± 2.14	0.007	4.25± 2.99	5.39± 2.58	0.143
**ROS** ^**a**^	6.29±4.65	11.20±6.26	0.003	6.93± 5.48	7.11± 3.77	0.895

CDAI: Clinical Disease Activity Index; DAS28: Disease Activity Score-28; FN: Functional status; PS: Psychiatry status; PN: pain; JCTC: self-report joint count; PTGL: patient global health status; EX: exercise; MS: morning stiffness; CHG: change in health status last week; FT: fatigue; ROS: review of system.

### Reliability

Out of the 140 RA patients asked to participate in the study, 137 patients agreed to participate and were placed in the reliability group portion of the study. However, only 133 out of the 137 patients completed the second round of the MDHAQ-P questionnaire (97.1%).

The average ICC for the test-retest measurement was 0.865(95% CI: 0.809, 0.904) and 0.786 (95% CI: 0.698, 0.848) for the sum score of the items of FN and PS respectively. The Cronbach’s alpha coefficients were high for both PS (0.705) and FN (0.885), which showed acceptable reliability of MDHAQ-P. The correlation between the items of FN ranged from 0.68 to 0.22. The highest correlation was between item a “dressing up” and item e “washing and drying the body”, and the lowest correlation was between item a “dressing up” and item j “participate in recreational activities and sports”. Our analysis of each item correlation in FN with the total item score displayed that the elimination of the FN dimension items did not significantly change the Cronbach’s alpha, ranging from 0.86–0.89 (**Table 4**).

**Table 4.** Internal consistency and item analysis of FN items.

**Items**	**Corrected Item-Total Correlation**	**Cronbach’s Alpha if Item Deleted**	**Item-Total Correlation**
a	0.625	0.873	0.579
b	0.67	0.87	0.498
c	0.601	0.876	0.464
d	0.619	0.874	0.51
e	0.703	0.868	0.616
f	0.77	0.862	0.652
g	0.581	0.876	0.461
h	0.632	0.873	0.504
i	0.619	0.874	0.55
j	0.464	0.89	0.362

In the PS dimension, correlations of item k “good night’s sleep” with two items related to anxiety and depression were 0.24 and 0.43, respectively. The correlation coefficients were less than the correlation between the two other items (0.65). By removing the item concerning sleep from the PS dimension, Cronbach’s alpha increased to 0.79 (**Table 5**).

**Table 5.** Internal consistency and item analysis of PS items.

**Items**	**Corrected Item-Total Correlation**	**Cronbach’s Alpha if Item Deleted**	**Item-Total Correlation**
k	0.372	0.787	0.186
l	0.534	0.599	0.423
m	0.685	0.392	0.499

## DISCUSSION

This study is the first translation and cross-cultural adaptation research conducted on the MDHAQ questionnaire in Iran. Rheumatic diseases such as RA have major effects on both the mental and physical health status of patients.^1,3,4^ MDHAQ is one of the latest versions of questionnaires used for rheumatology diseases.^2^ This study was designed to validate the MDHAQ-P as a rheumatologic assessment tool for Iranian patients with RA to evaluate qualitative factors regarding quality of life and determine the severity of the disease. The results showed that the Persian version of MDHAQ-P has acceptable validity and reliability for use in busy rheumatology clinics among Persian-speaking RA patients. In addition, the high response rate in our study indicates that the questionnaire was easy to complete and comprehend. RAPID3 and its components had a strong correlation with the Persian version of HAQ in our study. The HAQ has been used in other studies for evaluation of the convergent validity of different versions of MDHAQ.^11,22,23^ The FN item had a strong correlation with HAQ-DI in our study. These findings are consistent with the Chinese and Arabic versions of the MDHAQ.^11,22^ PS, CHG, FT, and ROS had a weak correlation with the Persian version of the HAQ. This is probably due to the contents of HAQ which focus more on disability and the functional dimension of rheumatologic diseases.^24^

The correlation between MDHAQ-P dimensions and clinical activity indexes of RA was also evaluated in our study. DAS28 and CDAI are the most frequent indices for the measurement of RA activity.^25^ MDHAQ is a self-reporting patient questionnaire, and unlike DAS28 and CDAI, there is no need for the clinician to complete.^2^ Some studies suggested RAPID3 as an alternative for CDAI and DAS28. Therefore, the amount of time required to visit the patients, especially in busy rheumatology clinics, can be reduced.^26,27^ CDAI had strong correlations with RAPID3 (rho= 0.838) and its components in our study. There was a moderate correlation between DAS28 and RAPID3 (rho= 0.439). In Pincus and his colleagues’ survey, RAPID3 was correlated with DAS28 (rho= 0.657) and CDAI (rho= 0.738) in 285 RA patients.^18^

In Young Song’s study containing 156 RA patients, the correlation coefficients were 0.701 for DAS28 and 0.843 for CDAI, respectively.^22^ DAS28 had a moderate correlation with RAPID3 in our study. This difference is probably due to the sample size of our study in comparison to the previously mentioned studies. In addition, since ESR is one of the components of DAS28, and age is an important factor that affects the level of ESR, considering the age group in DAS28 scoring may be useful.^28,29^

The EX dimensions did not correlate with either HAQ or clinical activity indexes. The frequency of physical activity is affected not only by the disease activity but is also influenced by culture, economic status, habits, and motivation.^30, 31^ 74% of the patients in this study did not exercise regularly. It seems that other factors besides the disease affect this dimension and further research may be needed for the reformulation of this dimension in the future.

The ICC of MDHAQ-P in the test-retest was acceptable in the FN (0.865) and PS (0.786) dimensions of our study. The ICC of the Finnish version of the MDHAQ was 0.93 and 0.84 for FN and PS, respectively.^23^ In the Swedish version of the MDHAQ, ICC was 0.85 for FN and 0.79 for PS.^10^ One of the reasons for these differences may be the test-retest intervals, which ranged from 48 hours to four weeks in the various studies.^10,11,23,32^ Another reason can be the differences in questions whose meanings were lost in translation.

Internal consistency of MDHAQ-P was strong in the FN (0.885) and PS (0.705) dimensions. By removing the item regarding quality of sleep, Cronbach’s alpha of PS dimension increased to 0.787. This finding was also present in other versions of MDHAQ.^10, 22^ Changing the scoring scale from 0–3.3 to 0–10, or removing this item from the PS dimension, can be useful for increasing the internal consistency of this dimension.^23^

One of the limitations of our study was the limitation of geography. Our centre is one of the referral centres in the south of the country and most of the subjects were residents of the south of the country, so the information and opinions obtained from patients living in other areas were limited. This could be developed in future research. Validation of the MDHAQ-P in other rheumatologic diseases could be considered an area of interest in future research studies.

In conclusion, the Persian form of MDHAQ is a reliable, applicable, and valid tool for evaluating the health status, and physical function, following the progress and outcome of RA patients in rheumatology clinic in Iranian RA patients. Further research and more evidence are needed for the modification of the questionnaire based on different races and dialects of the Iranian population in different regions.

## Data Availability

The datasets used and analysed during the current study are available from the corresponding author on a reasonable request basis.
